# Seventy Years Ago

**Published:** 1915-12

**Authors:** 


### SEVENTY YEARS AGO.

### Extracts from the Buffalo Medical Journal of 1845

---

**Twins at Different Stages of Development.** Dr. H. N. Loomis of Buffalo reports dead twin boys, at 8th month in a primipara. One was of corresponding development, the other of that of a foetus of the fifth month.

---

**Removal of 17 Inches of Small Intestine and Recovery.** Dr. Brigham, Superintendent of the Utica State Lunatic Asylum reports in Am. Jour. of Med. Sci., self-mutilation by a woman. Supposing the case to be necessarily fatal, Dr. Buttolph, the assistant physician, simply returned the end of the gut, the other end having spontaneously re-entered the abdomen, and stitched the abdominal wound. Complete recovery occurred, with spontaneous passage of bowels on 33d day.

---

**Phrenology.** Notice of pamphlet, containing lecture by Dr. Hamilton against Phrenology.

---

**First Intention.** Dr. F. H. Hamilton, in a series of articles on ''An'' European Tour, notes that Roux occasionally experiments in union by first intention after amputations, with some successes, ''but I should do him a great injustice to say that he has yet adopted this English principle as an established practice.''

---

**Dextrine Bandages for Simple Fractures.** Dr. Hamilton observes that Velpeau uses this for all simple fractures and questions whether it has any advantages over starch, used by Suetin. He condemns both.

---

**Lithotomy.** Dr. Alden S. Sprague reports a case in a French boy aged 11, who had been given much Burgundy in early childhood. Perineal section on a staff in the bladder was employed. Owing to the struggles of the patient, the rectum was wounded. The stone was reddish brown, 1x1½ inch. No ligatures were needed. The bladder was rinsed with warm milk and water and drained for two days. Urine passed partially by urethra on the 10th day and completely after the 15th. Liquid faeces were also passed by urethra for a short time. Healing complete on 22d day.

---

**Cancer a Local Affection.** Dr. Hamilton states that Velpeau, Roux, Blandin, Gerdy and Lisfranc regard cancer as originally local and that they remove many cases of scirrhous and open cancer which American surgeons would hesitate to touch.

---

**Anaplasty.** Dr. F. H. Hamilton discusses general principles of plastic operations and reports successful cases of blepharoplasty, cheiroplasty and rhinoplasty.

---

**Statistics of Medical Graduation and License for New York State.** College of Physicians and Surgeons, 37; University of New York, 92; Albany Medical College, 16; Geneva Medical College, 46; total 191. Seventeen county societies reported only 8 licentiates.

---

**Typhoid Epidemic.** Review of report by Dr. Austin Flint (1st) in Am. Jour. Med. Sci. In September, 1843, a young man from Massachusetts stopped at a tavern in N. Boston, Erie County, N. Y. He had symptoms of typhoid and died. 28 cases, 10 fatal, resulted in a population of 43. An autopsy with typic findings is included in the report. The fever was identified with the typhoid of New England and the reviewer, G. N. B. (Burwell) remarks that he has had 7 or 8 undoubted cases in 16 months. The paucity of literature is deplored and it is distinctly recognized that purgatives should not be used except at the commencement of the disease, that "an improper bleeding, emetic or a dose of physic even, ill-timed may cost the patient his life," also that the fever must run its course.

---

**Report of the Wounded at the Battle of Okee-Chobee, Fla.,** 25th December, 1839. A full report of wounds is contributed by Surgeon R. C. Wood of the Buffalo Barracks. Reference to this may be of interest to military surgeons.

---

**Consumption Considered Contagious** by all the people of the south of Italy and by many respectable physicians. ibid.

---

**Pellagra.** Dr. F. H. Hamilton, in his Notes of An European Tour, acknowledges the courtesy of Dr. Capelli of Milan in demonstrating cases. The symptomatology is graphically stated, the prognosis is almost hopeless, the causes are considered the hot, damp climate, poverty and especially the use of Indian corn made into a sour bread, and the use of sour wines. The treatment is plain, nutritious diet and baths, the last being especially emphasized.

